# Cortical Reorganization Is Associated with Surgical Decompression of Cervical Spondylotic Myelopathy

**DOI:** 10.1155/2015/389531

**Published:** 2015-11-02

**Authors:** Andrew Green, Priscilia W. T. Cheong, Stephanie Fook-Chong, Rajendra Tiruchelvarayan, Chang Ming Guo, Wai Mun Yue, John Chen, Yew Long Lo

**Affiliations:** ^1^Duke-NUS Graduate Medical School, 8 College Road, Singapore 169857; ^2^Department of Neurology, Singapore General Hospital, Singapore 169608; ^3^Department of Clinical Research, Singapore General Hospital, Singapore 169608; ^4^Department of Neurosurgery, National Neuroscience Institute, Singapore General Hospital, Singapore 169608; ^5^Department of Orthopaedic Surgery, Singapore General Hospital, Singapore 169608; ^6^Department of Neurology, National Neuroscience Institute, Singapore General Hospital, Singapore 169608

## Abstract

*Background*. Cervical spondylotic myelopathy (CSM) results in sensorimotor limb deficits, bladder, and bowel dysfunction, but mechanisms underlying motor plasticity changes before and after surgery are unclear. *Methods*. We studied 24 patients who underwent decompression surgery and 15 healthy controls. Patients with mixed upper and lower limb dysfunction (Group A) and only lower limb dysfunction (Group B) were then analysed separately. *Results*. The sum amplitude of motor evoked potentials sMEP (*p* < 0.01) and number of focal points where MEPs were elicited (*N*) (*p* < 0.001) were significantly larger in CSM patients compared with controls. For Group A (16 patients), sMEP (*p* < 0.01) and *N* (*p* < 0.001) showed similar findings. However, for Group B (8 patients), only *N* (*p* = 0.03) was significantly larger in patients than controls. Group A had significantly increased grip strength (*p* = 0.02) and reduced sMEP (*p* = 0.001) and *N* (*p* = 0.003) after surgery. Changes in sMEP (cMEP) significantly correlated inversely with improved feeding (*p* = 0.03) and stacking (*p* = 0.04) times as was the change in number of focal points (NDiff) with improved writing times (*p* = 0.03). Group B did not show significant reduction in sMEP or *N* after surgery, or significant correlation of cMEP or NDiff with all hand function tests. No significant differences in *H* reflex parameters obtained from the flexor carpi radialis, or central motor conduction time changes, were noted after surgery. *Discussion*. Compensatory expansion of motor cortical representation occurs largely at cortical rather than spinal levels, with a tendency to normalization after surgery. These mirrored improvements in relevant tasks requiring utilization of intrinsic hand muscles.

## 1. Introduction

Cervical spondylotic myelopathy (CSM) is one of the most common causes of spinal cord dysfunction in older individuals [1–3]. CSM is a chronic and progressive disease resulting from degenerative changes in the spine that gives rise to cord and nerve root impingement by osteocartilaginous elements [4]. These lesions cause much morbidity in patients, including sensorimotor limb deficits, bladder, and bowel dysfunction. Many patients with CSM are treated surgically with the hope of preventing further neurological deterioration or achieving some functional recovery [4, 5]. However, the physiological mechanisms underlying the recovery of motor function after CSM surgery are poorly understood.

Evidence in the medical literature suggests that the improvement of motor function after surgical decompression in CSM patients may occur via synaptic changes and dendritic sprouting in the cortical and spinal cord neuron pools [6, 7]. Firstly, the natural process of functional recovery without medical intervention in many pathological situations involves plasticity changes in the motor cortex. For example, transcranial magnetic stimulation (TMS) studies in stroke patients have shown that motor recovery is associated with improved corticospinal conduction as well as cortical reorganization [8, 9]. This recovery process is not limited to the event of cortical damage. In fact, Nishimura et al. [10] have demonstrated that functional reorganization in bilateral premotor and primary motor areas took place after lateral corticospinal tract transection at the cervical level in macaque monkeys. These plasticity changes in the motor cortices were associated with restoration of skilled finger movements. Similarly, neuroimaging studies in humans have affirmed that rapid cortical and subcortical reorganization are a common occurrence after spinal cord injury and/or myelitis [11–13]. In patients with cervical myelitis, robust changes within the sensorimotor cortex were inversely correlated with the severity of the spinal cord damage [11]. Taken together, these findings strongly indicate the importance of cortical reorganization in sensorimotor function improvement after spinal cord injury.

Without medical intervention, the natural recovery process following spinal cord compression is slow and largely depends on the extent of the injury sustained [12, 14, 15]. A number of trials have shown that CSM patients treated with decompression surgery experienced neurological improvements and, as such, surgical intervention is often recommended in moderate and severe CSM cases [4, 13]. In addition, serial functional magnetic resonance imaging (fMRI) studies have captured the evolving changes in the cerebral cortex in CSM patients following surgical decompression [7, 16]. However, no study thus far has compellingly shown the direct relationship between cortical plasticity and the degree of motor improvement after spinal cord injury.

An emerging modality used to study functional organization in the human motor cortex is TMS [2, 8, 17]. It is a noninvasive tool that measures conduction in the descending corticospinal pathways and is capable of rapidly evaluating output assessing the functional organization and reorganization of the human motor cortex [8, 18, 19]. Within this framework, we aim to investigate the association between cortical reorganization and motor function improvement after cervical decompression surgery. We hypothesize that a correlation exists between the plasticity in the cortex and improvement in motor function scores (as measured by the Modified Japanese Orthopaedic Association Score) [2, 17] and detailed tests of hand function [20] in moderate-to-severe CSM patients four months after spinal cord decompression surgery. Secondarily, we investigate compensatory motor cortex representation changes in CSM patients in relation to healthy controls.

## 2. Methods

### 2.1. Subjects

With ethics committee (Singapore General Hospital ethics committee) approval, patients presenting with clinical features of CSM of at least 6 months' duration who were listed for spinal cord decompression surgery were recruited with informed consent obtained. We excluded patients with suspected traumatic spinal injury, or any underlying medical or neurological condition which may confound electrophysiological findings. MRI of the cervical spine was performed in all patients within 1 month before surgery. No physiotherapy sessions were scheduled for these patients after surgery. Every recruited patient underwent TMS and motor function testing 1 month prior to and 4 months after surgery. The operation is usually anterior laminectomy of the cervical spine, or any additional procedure stabilization. We also recruited healthy controls for comparison.

### 2.2. Transcranial Magnetic Stimulation

TMS mapping of the left hemisphere was performed using a Medtronic (Medtronic Corporation, USA) figure-of-eight-shaped C-B60 coil with 7 cm internal diameter connected to a Medtronic R8 unit generating a peak magnetic field of 2.2 Tesla. The coil was placed tangentially over the skull with the handle pointing backwards and perpendicular to the direction of the central sulcus at approximately 45 degrees to the midline to evoke an anteromedially directed current in the brain.

The vertex, designated as intersection of the interaural line and the nasion-inion connection, was used as an anatomical landmark for finding the optimal position (hotspot) for eliciting motor-evoked potentials (MEPs) from the right first dorsal interosseous (FDI). This is defined as the position with the lowest stimulation intensity needed to elicit an MEP. At the hotspot, the resting motor threshold (rMT) is determined as the position where the lowest TMS intensity will elicit an MEP at a vertical gain of 50 *μ*V/division for 5 out of 10 stimulations. Once these procedures were completed, the hotspot is placed as the centre of a square-shaped 25-position grid drawn along both the anteroposterior and the mediolateral axes on the subject's head. Each point is spaced 1 cm apart from its adjacent position. The map for the right FDI was then obtained by stimulating each point of the grid lying over the motor strip. For each scalp position, we recorded the mean of MEP amplitudes evoked by 5 stimulations at 110% of the rMT. During the recording, which required EMG silence, muscular activity was constantly monitored. MEPs were amplified, filtered, and recorded on a Medtronic Keypoint electromyography machine with a band pass of 20 to 2000 Hz for analysis. Continuous EMG and sound monitoring ensured only nonfacilitated responses will be included for analysis.

TMS parameters obtained were the sum amplitude of MEPs (sMEP) of the entire 25-point grid and number of positions (*N*) where MEPs could be elicited. We also computed the difference in sMEP (cMEP) and *N* (NDiff) before and after surgery in each patient. For comparison, healthy age-matched controls had similar TMS motor mapping performed.

To better ascertain if corticospinal excitability changes occur at the spinal or supraspinal levels, *H* reflexes were obtained from the right flexor carpi radialis by stimulating the median nerve at the elbow level. Both *H* amplitude and *H*/*M* ratios were noted, where *M* referred to amplitude of the flexor carpi radialis compound muscle action potential, as described previously [21, 22].

Central motor conduction times (CMCT) were also obtained from both upper and lower limbs in all patients before and after surgery. CMCT methodology was in accordance with previously published studies by the same authors [21, 22].

### 2.3. Motor Function Testing

Apart from clinical history and physical examination, each patient's motor function was quantitatively assessed using Modified Japanese Orthopaedic Association Score Scale (mJOAS) [17, 23] and Jebsen test of hand functions (JHFT) [20]. The tests were done at baseline and 4 months after operation and documentation was by an investigator who did not perform the surgical procedure.

### 2.4. Data Analysis

As CSM can result in exclusively upper limb or lower limb complaints as well as mixed upper and lower limb features, we separated patients into two groups. In Group A, all had mixed upper and lower limbs features, but patients in Group B had features exclusive to the lower limbs, in line with the mJOAS described above. None of the patients experienced sphincter disturbances.

Statistical calculations were made using SPSS for Windows software. The Wilcoxon Signed-Rank test was used to compare means and Spearman correlation coefficient was employed to examine the relation between MEP characteristics and functional changes in patients after surgery. A *p* value of <0.05 denoted statistical significance.

## 3. Results

All 24 patients (16 males, 8 females, mean age ± SD: 58.2 ± 11.5) were right handed as were the 15 healthy age-matched control subjects.

mJOA scores were significantly improved after surgery for all patients (*p* = 0.03).

For all patients, we found that sMEP (*p* = 0.0014) and *N* (*p* = 0.0008) were significantly larger preoperatively.

The sMEP (*p* = 0.012) and *N* (*p* = 0.0008) were significantly larger in preoperative CSM patients compared with healthy controls.

Separately, for Group A (16 patients), sMEP (*p* = 0.003) and *N* (*p* = 0.001) were also larger than healthy controls. However, for Group B (8 patients), only *N* (*p* = 0.0026) was significantly larger than healthy controls.

Postoperatively, no significant differences in sMEP for Group A (*p* = 0.08) or Group B (*p* = 0.796) were found compared with controls. However, for *N*, Group A (*p* = 0.01) was still significantly larger than healthy controls. This was not seen in Group B (*p* = 0.12) compared with healthy controls.

For Group A, we found significantly reduced sMEP (*p* = 0.001) and *N* (*p* = 0.003) after surgery. In addition, significantly increased grip strength (*p* = 0.02) and improved time for picking small objects (*p* = 0.04) were noted. Specifically, cMEP, in terms of reduction of sum of MEP amplitudes after surgery, significantly correlated with improved feeding (*r* = 0.25, *p* = 0.03) and stacking (*r* = 0.52, *p* = 0.04) times. NDiff in terms of reduction after surgery in number of excitable positions where MEPs were elicited significantly correlated with improved writing times (*r* = 0.48, *p* = 0.03).

For Group B, there was no significant reduction in sMEP or *N* after surgery, and no significant correlation was found for cMEP or NDiff with all hand function tests.

We did not find significant differences in CMCT from all 4 limbs and *H* reflex parameters before and after surgery.

Table 1 summarizes study results of patients and controls.

**Figure 1:**
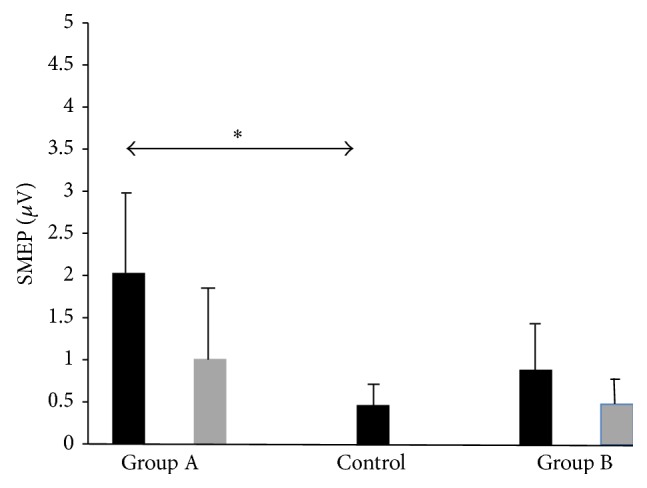
sMEP findings graphically. Asterisks denote statistical significance. Preoperative bars are black and postoperative bars are grey. sMEP, sum of MEP amplitudes in mV in vertical axis. Horizontal axis depicts patient and control groups.

Figures 1 (sMEP) and 2 (*N*) depict MEP mapping findings graphically. Asterisks denote statistical significance. Preoperative bars are black and postoperative bars are grey.

**Figure 3:**
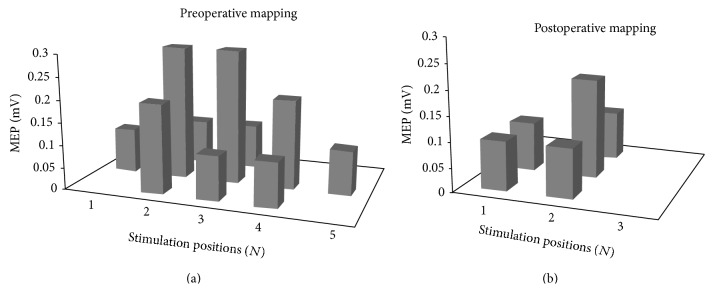
Schematic diagram depicting motor output mapping of a patient in Group A. In the preoperative grid, sMEP is 1.7 mV as sum total of 10 stimulation positions eliciting an MEP (*N* = 10). Postoperatively, sMEP was reduced to 0.7 mV and *N* to 5. sMEP, sum of MEP amplitudes in mV.

Figure 3 is a schematic diagram depicting motor output mapping of a patient in Group A preoperatively and postoperatively.

## 4. Discussion

In the first TMS study of this nature to our knowledge, we sought to provide a vital connection between existing studies using functional imaging and the recovery process after decompression surgery in CSM.

Early imaging studies in CSM have focused on morphological changes in operated CSM patients. Fukushima et al. [24] showed that good functional outcome after surgery is correlated with a minimum re-expanded cord area in 55 patients. Baba et al. [13] separately studied 56 patients and concluded that early postoperative cord expansion reflected improved clinical status and suggested that this may be due to enhanced “intracord plasticity.” However, these studies did not utilize electrophysiology as a bridge to explain clinical and morphological changes.

The advent of functional imaging, including PET and fMRI, provided new information on brain remodelling by virtue of blood flow changes. In terms of spinal cord lesions, traumatic spinal cord injury (SCI) is known to induce expanded brain activation towards the leg areas, thalamus, and cerebellum as seen in PET studies [25]. In a separate group of 6 SCI patients, fMRI showed initial decrease and then increase of activation of sensorimotor areas [26], reflecting the dynamic response of brain function probably as a compensatory mechanism. In terms of morphology, complete SCI patients exhibited reduced gray matter volume in the primary motor, medial prefrontal, cingulate, and cerebellar cortex, in addition to diffusion tensor imaging (DTI) changes in cortical [27, 28] and brainstem motor areas [29]. When interpreting these findings, it should be noted that SCI may differ in onset, chronicity, and extent, which may in turn affect the cortical or subcortical changes observed.

Specifically for CSM, few studies have been published to date addressing fMRI changes before and after decompression. Holly et al. [16] found evidence of expanded cortical representation of the affected arm. Following surgery, distinct reorganization of this representation was seen but not in any consistent pattern. In a further 8 patients studied by the same group [7], postoperative activations of sensorimotor areas normalized to become similar to healthy controls after CSM decompression surgery. In contrast, a study by Duggal et al. [29] in CSM patients demonstrated a larger volume of activation within the precentral motor areas and reduced volume of activation in the postcentral areas before operation. Postoperatively, continued enlarging volumes of activation were noted in both these areas regions of interest. In summary, while most fMRI evidence points to increased activation of motor areas in CSM before operation, the findings postoperatively did not indicate a uniform pattern of activation. The underlying reasons remain unclear, and further investigation, particularly in conjunction with electrophysiological or neurobiological methods, is justified.

Neurobiological evidence certainly exits with regard to the axonal sprouting and contacting of propriospinal neurons in animal experiments after transection of corticospinal projection to the hind limbs [30]. Additionally, brain-derived neurotrophic factor (BDNF) and neurotrophin-3 delivered to corticospinal neurons resulted in increased collateral sprouting and contacts with propriospinal neurons [31, 32]. In somatosensory deprived rats by thoracic cord transections, upregulation of BDNF and decrease of gene activity for Nogo receptor were also demonstrated [33]. In another experiment, intrathecal Nogo receptor antagonist promoted growth of corticospinal axons in lesioned rats [34]. Collectively, these findings provide evidence to further support clinical, electrophysiological, and imaging data, suggesting modulation of neuroplasticity in response to spinal cord lesions.

In summary, TMS mapping of the motor cortex is well recognized to reflect functional plasticity of cortical outputs topographically [35]. The technique has been utilized to investigate cortical reorganization with training tasks, stroke rehabilitation, and peripheral limb amputation. While there is no universally standardized technique for motor mapping, MEP amplitudes and number of excitable sites [36–38] over a grid area [39] have been used extensively as mapping parameters. The methodology has been found to be robust and stable over time [40].

Before decompressive surgery, increased cortical representation of intrinsic hand muscle compared to normal controls is not unexpected and likely reflects an inherent compensatory mechanism in response to cord compromise. The observation is corroborated by functional imaging in spinal cord injury [25, 26] and CSM [16] as well as animal models [30–33]. However, postoperatively, the tendency to normalization of motor representation is less well understood and inconsistent [29] but may be best explained in relation to recovery from chronic partial spinal cord injury. Like spinal cord injury, CSM can be heterogeneous, and it may be crucial but difficult to distinguish natural recovery compensatory mechanisms and that due to therapeutic intervention, such as motor training. Even in the present study whereby all patients do not receive physiotherapy, postoperative motor activity can be different for each patient, and standardization will be challenging over a 4-month period. Additionally, in the recovery period, the extent of synaptic transmission and reorganization is dependent on time after the initial insult to the spinal cord [41] as well as the variable degree of residual spinal cord atrophy [42]. While our findings point to reduction and normalization of motor representation 4 months after surgery, the findings cannot be reliably corroborated with published imaging studies in view of differences in follow-up duration and lack of a repeat MRI in most studies to ascertain cord atrophy.

In CSM, compression of descending corticospinal tracts results in desynchronization of I-wave volleys evoked with single pulse TMS of the primary motor cortex. The MEPs obtained can be used to calculate the CMCT by subtracting the peripheral conduction time. CMCT is more sensitive measure of corticospinal dysfunction in CSM than somatosensory evoked potentials [43–45] and can be utilized for the presurgical evaluation of CSM patients in the clinical setting [17]. In a prospective study of 141 CSM patients, excellent correlation of MRI with CMCT in terms of sensitivity and specificity was demonstrated [22]. Another prospective study of 241 patients found that TMS parameters had 98% sensitivity and specificity for mild cord compression, suggesting that TMS can be employed as a screening tool in CSM before MRI [2].

Noteworthy though, we did not find significant CMCT changes before and after surgery in all 4 limbs, despite motor cortex excitability modulation evident with cortical mapping as well as improvement in hand function in relation to MEP changes. In line with these observations, modulation of the ability to facilitate horizontal rather than vertical synaptic connections would be the most likely underlying mechanism at play. As TMS largely stimulates cortical neurons in a transsynaptic fashion [46], motor mapping with TMS will likely yield the most valid information in terms of plasticity changes. To our knowledge, this has only been studied in the context of SCI. Streletz et al., using serial motor mapping of C5 to C6 SCI patients, showed that enlarged contralateral biceps representation was present as early as Day 6 after injury [47]. In contrast, a separate study of 22 SCI patients using TMS did not show significant map changes after injury [48]. Similar to functional imaging, it can thus be appreciated that TMS motor mapping after cord dysfunction also did not yield findings with uniform characteristics. To our knowledge, studies of this nature have not been performed in CSM pre- or postoperatively. CMCT is the most frequently used and sensitive electrophysiological parameter to evaluate CSM clinically, and its methodology is fairly standardized across clinicians and researchers. CMCT evaluates motor cortex to anterior horn cell conduction and reflects integrity of rapid, direct descending pyramidal connections to the same intrinsic hand muscle (FDI) used for motor mapping in the present study. This further adds to the validity of our observations that lack of CMCT changes postoperatively implies modulation of the ability to facilitate horizontal rather than vertical synaptic connections as the most likely underlying mechanism at play.

The lack of *H* reflex modulation despite significant TMS mapping changes suggests that supraspinal rather than spinal mechanisms are predominant in driving plasticity after surgery. These findings are also in line with our previous impression that horizontally orientated cortical elements are largely responsible with observed TMS motor mapping changes. Modulation of the *H* reflex is well known to be reflective of changes in spinal excitability [49]. It has been used to assess spinal interneuronal excitability at rest and even during movement [50] as well as in combination with TMS efficaciously [51]. Although the *H* amplitudes and *H*/*M* ratios [51] are largely contributed by monosynaptic 1a excitation of spinal motor neurons [52], other mechanisms, including reciprocal and 1b inhibition, are known to modulate *H* reflex characteristics. Thus, it is imperative that recording conditions must be standardized to allow for a relaxed patient in quiet experimental conditions, delivering fixed stimulation parameters.

In the light of current knowledge outlined above, it is imperative that our findings can be applied to elucidate modulation of cortical motor control mechanisms in CSM. Based on comparison with healthy controls and within each patient, compensatory expansion of the hand area, in terms of magnitude and spatial representation of cortical excitability postoperatively, is evident. These observations are further strengthened by findings that, for Group A patients, both magnitude and spatial characteristics were larger than controls, whereas for Group B, only spatial characteristic were. This may be related to Group A patients having relatively more upper limb motor deficits compared with Group B, hence, driving enhanced cortical compensatory representation [53]. Furthermore, postoperatively, reduction in magnitude and spatial characteristics of cortical excitability were seen only in Group A, reflecting, for similar reasons, compensatory changes more specific to upper limb functional deficits, compared to Group B patients with lower limb dysfunction exclusively.

We next examined cortical excitability modulation in relation to the functional relevance of these changes. In terms of objective hand function tests, significantly increased grip strength and reduced lifting time for small objects rather than the other tests likely reflected improved direct projections for intrinsic hand musculature. However, significant correlation of changes in magnitude of cortical excitability for both feeding and stacking objects also likely reflects participation of more proximal muscles needed for these tasks which were modulated in terms of horizontal placed connectivity postoperatively. Similarly, spatial changes in terms of number of excitable sites during TMS correlating with writing tasks also reflected functional cortical participation for both intrinsic muscle and wrist action, corroborating the experimental design and TMS both evaluating predominantly motor representation of distal muscles performing more finely skilled tasks.

All these observations, again, were seen exclusively in Group A patients, and all hand function tests were designed to evaluate the upper limb only. It would be interesting to compare our findings with the only fMRI study to date incorporating hand function tests [7]. In the 3-finger pinch tests, pinch-related activation volume in the ipsilateral sensorimotor cortex and the magnitude of activation in the contralateral dorsal premotor cortex evolved linearly across time after surgery, along with wrist extension-related activation magnitude in the contralateral supplementary motor area. However, in contrast to our findings which suggested reduction and return to normalcy of cortical excitability after surgery, there was no unidirectional change noted. The exact reasons are unclear, but the two studies employ different evaluation methods, as well as nonidentical hand function tasks which may partially explain differential results.

It is noteworthy that current knowledge may be limited by several factors. For fMRI, tasks are often limited to motor imagery rather than actual muscle activation due to the presence of movement artefacts. For electrophysiological studies, however, both resting and active tasks can be studied. In an event when both functional imaging and TMS results must be combined, it should thus be noted that findings may not be directly comparable. Overall, published studies are usually small in subject numbers, lacking in standardization of protocols and serialization of data. These deficiencies should be addressed in larger future studies of a similar nature.

In conclusion, we have demonstrated that compensatory expansion of motor cortical representation with a tendency to normalization after surgery occurs largely at cortical rather than spinal level. Cortical plasticity modulation mirrored improvements in relevant tasks requiring utilization of predominantly distal hand muscles. These findings have important implications with regard to the understanding and rehabilitation of patients with lesions involving the cervical spinal cord.

## Figures and Tables

**Figure 2:**
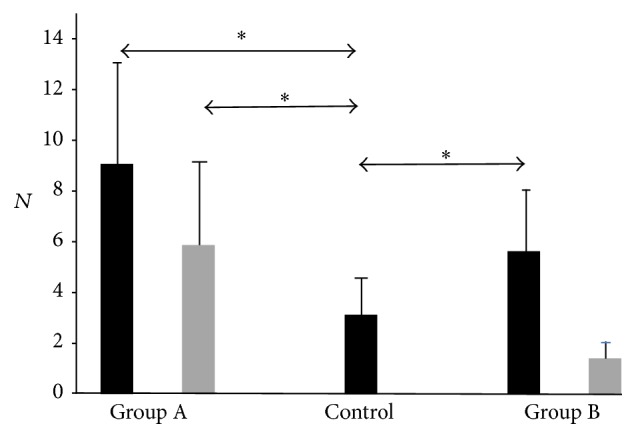
*N* findings graphically. Asterisks denote statistical significance. Preoperative bars are black and postoperative bars are grey. Vertical axis depicts number of excitable positions where MEP is elicited (*N*). Horizontal axis depicts patient and control groups.

**Table 1:** Summary of experimental results in all patients.

	Preoperative		Postoperative		Significance
mJOA	12.7 (2.81)		13.81 (3.1)		*p* = 0.03^*∗*^
sMEP	1.64 (1.88)		0.82 (0.89)		*p* = 0.0014^*∗*^
*N*	7.86 (3.93)		5.22 (2.58)		*p* = 0.0008^*∗*^

Group	A	B	A	B	

sMEP	2.03 (1.54)	0.89 (0.55)	1.01 (1.05)	0.48 (0.31)	Group A (*p* = 0.003)^*∗*^ Group B (*p* = 0.65)
*N*	9.07 (4.00)	5.60 (2.80)	5.87 (2.95)	4.00 (1.41)	Group A (*p* = 0.0001)^*∗*^ Group B (*p* = 0.0026)^*∗*^
*Jebsen tests *					
Write	23.98 (25.49)	12.11 (5.84)	20.97 (20.08)	11.71 (6.10)	Group A (*p* = 0.60) Group B (*p* = 0.25)
Turn page	9.56 (9.15)	5.98 (2.22)	7.19 (3.53)	7.89 (4.86)	Group A (*p* = 0.17) Group B (*p* = 0.18)
Lift small object	11.43 (7.06)	9.65 (4.26)	9.19 (4.13)	9.54 (6.10)	Group A (*p* = 0.04)^*∗*^ Group B (*p* = 0.98)
Feed	13.20 (6.74)	13.06 (7.12)	12.03 (5.89)	11.38 (5.74)	Group A (*p* = 0.58) Group B (*p* = 0.12)
Stack	5.64 (6.43)	2.85 (2.17)	4.00 (6.13)	2.78 (1.07)	Group A (*p* = 0.31) Group B (*p* = 0.83)
Lift light can	4.23 (3.88)	4.69 (2.87)	4.21 (3.91)	4.56 (3.90)	Group A (*p* = 0.43) Group B (*p* = 0.65)
Lift heavy can	5.18 (2.34)	5.14 (3.34)	4.87 (2.02)	4.89 (3.33)	Group A (*p* = 0.34) Group B (*p* = 0.62)
*Other tests *					
9-hole peg	68.36 (41.78)	52.63 (30.00)	57.39 (28.63)	59.77 (34.12)	Group A (*p* = 0.26) Group B (*p* = 0.14)
Tap	68.63 (12.09)	70.43 (10.83)	69.79 (4.57)	74.79 (8.13)	Group A (*p* = 0.92) Group B (*p* = 0.14)
Pinch grip strength	15.55 (7.46)	20.86 (3.8)	17.87 (7.33)	22.64 (4.68)	Group A (*p* = 0.02)^*∗*^ Group B (*p* = 014)
*Electrophysiology *					
CMCT					
R UL	10.77 (3.22)	7.89 (2.34)	9.88 (2.16)	7.69 (2.87)	Group A (*p* = 0.23) Group B (*p* = 0.31)
L UL	11.65 (3.45)	7.99 (2.77)	10.45 (2.98)	7.62 (2.96)	Group A (*p* = 0.32) Group B (*p* = 0.45)
R LL	18.34 (3.98)	19.23 (4.11)	17.97 (4.06)	19.86 (4.68)	Group A (*p* = 0.64) Group B (*p* = 0.46)
L LL	19.11 (4.07)	19.25 (4.87)	18.78 (4.39)	20.12 (4.61)	Group A (*p* = 0.51) Group B (*p* = 0.48)

*H* amplitude	1.22 (0.53)	1.34 (0.45)	1.21 (0.47)	1.29 (0.51)	Group A (*p* = 0.51) Group B (*p* = 0.48)
*H*/*M*	0.82 (0.23)	0.91 (0.22)	0.77 (0.36)	0.86 (0.28)	Group A (*p* = 0.41) Group B (*p* = 0.57)

Mean values are indicated (standard deviation).

All hand function test results are in seconds.

In healthy controls, mean sMEP was 0.51 (0.28) and *N* was 3.36 (1.21).

CMCT: central motor conduction time (m/s); R: right; L: left; UL: upper limb; and LL: lower limb.

*H*/*M*: *H* amplitude (mV)/*M* amplitude (mV).

*∗* denotes statistical significance at *p* < 0.05.
